# Comparison of fully versus partially covered metal stents in endoscopic ultrasound‐guided hepaticogastrostomy for malignant biliary obstruction (with video)

**DOI:** 10.1111/den.14952

**Published:** 2024-11-28

**Authors:** Sung Hyun Cho, Seong Je Kim, Tae Jun Song, Dongwook Oh, Dong‐Wan Seo

**Affiliations:** ^1^ Department of Gastroenterology, Asan Medical Center University of Ulsan College of Medicine Seoul South Korea; ^2^ Department of Gastroenterology, Gyeongsang National University Hospital Gyeongsang National University School of Medicine Jinju South Korea

**Keywords:** bile duct, drainage, endosonography, gastrostomy, stents

## Abstract

**Background:**

Endoscopic ultrasound‐guided hepaticogastrostomy (EUS‐HGS) using a fully covered metal stent (FCMS) or partially covered metal stent (PCMS) is performed to manage unresectable malignant biliary obstruction (MBO) following unsuccessful endoscopic retrograde cholangiopancreatography. This study aimed to compare FCMS and PCMS for EUS‐HGS in patients with MBO.

**Methods:**

We reviewed the EUS database to analyze consecutive patients with MBO who underwent EUS‐HGS between November 2017 and March 2023. We performed a 1:1 matching using propensity score matching based on potential confounding factors. Stent patency, technical success, clinical success, adverse events, reintervention, and overall survival were assessed.

**Results:**

The technical success rate of EUS‐HGS was 92% (123/134). A total of 80 patients with technical success (40 FCMS, 40 PCMS) were selected after propensity score matching. The two groups showed similar rates of clinical success (90% vs. 88%; *P* = 0.999), early adverse events (15% vs. 20%; *P* = 0.556), late adverse events (18% vs. 33%; *P* = 0.121), reintervention (20% vs. 38%; *P* = 0.084), and median overall survival (4.1 months [95% confidence interval (CI) 2.6–5.5] vs. 3.8 months [95% CI 1.9–5.7]; *P* = 0.609). During follow‐up, the FCMS group showed higher patency rates (85% vs. 60% at 6 months; 76% vs. 43% at 12 months; *P* = 0.030).

**Conclusions:**

FCMS and PCMS for EUS‐HGS in patients with unresectable MBO showed similar rates of clinical success, as well as early and late adverse events. However, the FCMS group showed a higher cumulative stent patency rate compared to the PCMS group.

## INTRODUCTION

In patients with unresectable malignant biliary obstruction (MBO), endoscopic retrograde cholangiopancreatography (ERCP) with palliative metal stenting has been the primary therapeutic modality for biliary drainage.^1^, ^2^ Endoscopic ultrasound (EUS)‐guided biliary drainage (EUS‐BD) can be performed when ERCP fails to drain the obstructed bile duct, or when a trans‐papillary approach is not accessible. EUS‐BD has advantages over percutaneous transhepatic biliary drainage (PTBD) due to a lower rate of adverse events and reinterventions.^3^, ^4^, ^5^, ^6^, ^7^

EUS‐guided hepaticogastrostomy (EUS‐HGS) for draining the left intrahepatic duct (LIHD) in patients with unresectable MBO is well‐documented as EUS‐BD.^6^, ^8^, ^9^, ^10^, ^11^, ^12^ Recent guidelines also recommend EUS‐HGS after failed ERCP and/or PTBD for LIHD drainage in patients with unresectable MBO in high‐volume centers.^13^ As EUS‐HGS has become more popular, various types of dedicated stents for EUS‐HGS have been developed.^14^, ^15^, ^16^, ^17^, ^18^ In cases of MBO, self‐expandable metal stents (SEMS) are recommended for use in EUS‐HGS to prevent bile leakage.^19^, ^20^ The types of SEMS for EUS‐HGS include fully covered metal stents (FCMS) and partially covered metal stents (PCMS), but an optimal type of SEMS has not been widely accepted thus far. Dedicated PCMS was introduced to minimize adverse events associated with FCMS such as stent migration and side‐branch occlusion.^14^, ^16^, ^18^, ^21^ While dedicated PCMS for EUS‐HGS demonstrated favorable long‐term outcomes,^9^, ^16^ no studies to date have directly compared FCMS and PCMS for EUS‐HGS. Therefore, we aimed to compare the outcomes of FCMS and PCMS for EUS‐HGS in MBO.

## METHODS

### Patients

We retrospectively reviewed the EUS database at the Asan Medical Center to gather data on consecutive patients with unresectable MBO who underwent EUS‐HGS alone without the use of a bridging technique or antegrade stenting between November 2017 and March 2023. The decision regarding the type of metal stent between FCMS and PCMS was made at the discretion of the attending endosonographer. EUS‐HGS was performed as a rescue therapy when ERCP failed to drain the obstructed bile duct, or as the primary therapy when a trans‐papillary approach was not accessible due to surgically altered anatomy or duodenal obstruction. The exclusion criteria were as follows: (i) patients aged under 20 years; (ii) placement of a plastic stent for EUS‐HGS; or (iii) benign biliary stricture.

### Characteristics of the stent design

#### FCMS

**Figure 1:**
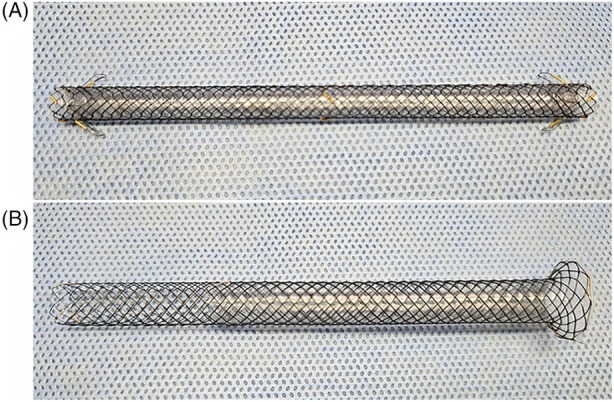
Design of the stents. (A) Fully covered self‐expandable metal stent. (B) Partially covered self‐expandable metal stent.

The FMCS (HANAROSTENT Biliary Flap [CCC] Dual Flap; MI Tech, Seoul, Korea) is constructed from nitinol wire and is fully covered with a silicon membrane (Fig. 1A). The stent has antimigrating anchoring flaps at both the proximal and distal ends. The FCMS used in this study had a diameter of 6 mm, and the stent length was sufficient to prevent stent migration (10 cm).

#### PCMS

The PCMS (Giobor; Taewoong Medical, Ilsan, Korea) has a 3 cm uncovered portion made of braided nitinol wire at the hepatic end and a 7 cm fully covered portion with a silicon membrane at the gastric end (Fig. 1B). The PCMS used in this study has a diameter of 8 mm and a length of 10 cm. The fully covered portion was positioned from the intrahepatic duct to the gastric lumen to prevent bile leakage or pneumoperitoneum.

### Procedures

**Figure 2:**
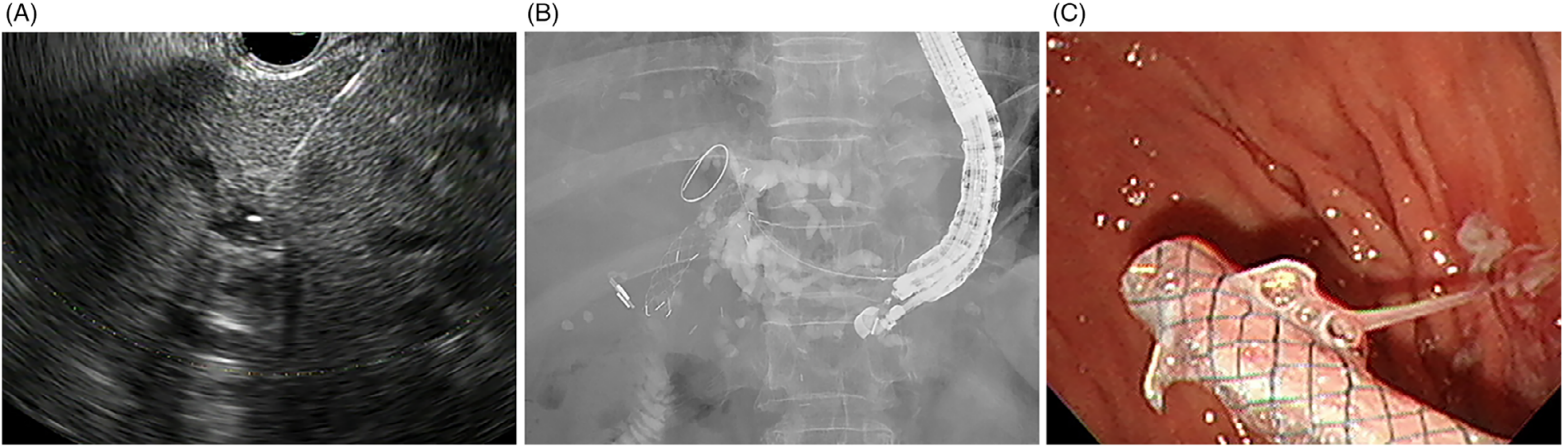
Endoscopic ultrasound‐guided hepaticogastrostomy. (A) Endoscopic ultrasound image showing the dilated left intrahepatic duct punctured by a 19G needle. (B) The fully covered metal stent (FCMS) was advanced through the guidewire and deployed using the intrascope release technique. (C) Duodenoscopic view showing the gastric ends of FCMS after successful deployment.

Before the needle puncture, a 0.025 inch guidewire (VisiGlide‐2; Olympus, Tokyo, Japan) was preloaded into a 19G needle with three‐way stopcock following the prefilling the 19G needle with normal saline. A 19G needle (EUSN‐19‐T; Cook Endoscopy, Winston‐Salem, NC, USA) punctured the dilated LIHD through the gastric wall (Fig. 2A). After the guidewire was advanced into the bile duct, contrast media was then injected into the LIHD through the needle with three‐way stopcock to confirm biliary access. A 4 mm biliary balloon dilator (REN; Kaneka Medical, Osaka, Japan) was used to dilate the fistula tract. The FCMS or PCMS was advanced toward the LIHD over the guidewire (Fig. 2B). The intra‐scope channel stent release technique was used to deploy the metal stent (Fig. 2C).^22^, ^23^ The adjunctive techniques following deployment, such as clipping, were not performed. The detailed technique of EUS‐HGS is described in Data S1 and Video S1.

### Follow‐up

The follow‐up laboratory tests and simple abdominal radiographs were performed every month after the procedure or whenever recurrent cholangitis or other stent‐related adverse events occurred. An additional computed tomography scan was performed to assess stent malfunction in patients suspected of recurrent cholangitis or adverse events. The scheduled stent replacement was not regularly performed because patients with unresectable MBO had a limited life expectancy, and frequent endoscopic procedures could be burdensome for this study group.

### Definition of outcomes

The primary outcome of this study was the cumulative stent patency rate at 6 and 12 months. The secondary outcomes included technical success, clinical success, early and late procedural adverse events, reinterventions, and overall survivals.

The location of biliary obstruction (proximal or distal MBO) and a history of chemotherapy were potential confounding factors in the propensity score matching analysis. Distal MBO was defined as a biliary stricture located at least 2 cm away from the liver hilum (bifurcation).

Technical success was defined as the successful placement of the stent into the LIHD, ensuring adequate flow of bile and radiocontrast through the stent. Clinical success was defined as a reduction in serum bilirubin levels to less than 50% of the pretreatment level within 2 weeks after the procedure or the complete resolution of cholangitis.^13^ Procedure‐related adverse events were defined according to the lexicons of the American Society of Gastrointestinal Endoscopy for adverse events.^24^ These events were classified as early (within 2 weeks) and late (2 weeks or more) adverse events based on the time of presentation. Stent patency was defined as the interval between stent insertion and the occurrence of stent occlusion, change, removal, or dislodgement. In the analysis of cumulative stent patency, patients were censored at the time of their last follow‐up or death. Reintervention was defined as any endoscopic or interventional radiologic biliary procedure performed to manage the early or late adverse events, or clinical failure following EUS‐HGS.

### Statistical analysis

The results are presented as numbers with percentages or as the mean and standard deviation (SD). Comparisons were performed using the χ^2^‐test or Fisher's exact test for categorical variables, and Student's *t*‐test for continuous variables. The cumulative stent patency and cumulative overall survival were analyzed in all patients who achieved technical success using the Kaplan–Meier method, along with a log‐rank test.

To enhance comparability between the FCMS and PCMS groups, propensity score matching was performed. The propensity score was estimated using multiple logistic regression analysis, with variables including age, sex, age‐adjusted Charlson comorbidity index, indication for EUS‐HGS (primary or rescue EUS‐HGS), level of biliary obstruction (proximal or distal biliary obstruction), targeted LIHD (B2 or B3), and chemotherapy. The 1:1 greedy matching with a caliper of 0.2 SDs of the logit of the propensity score was performed. The absolute standardized mean differences were used to assess the balance, and all absolute standardized mean differences after matching were less than 0.1. *P*‐values less than 0.05 were considered statistically significant. All statistical analysis was performed using SPSS Statistics version 22.0 (IBM, Armonk, NY, USA).

## RESULTS

### Patient characteristics

**Figure 3:**
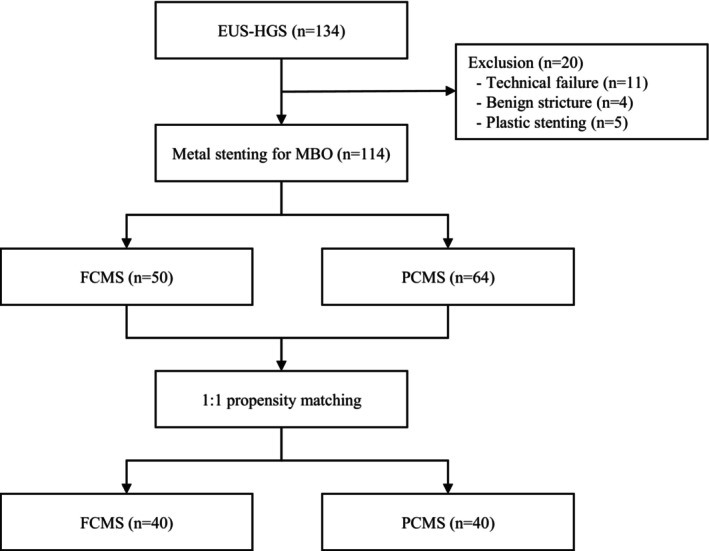
Flowchart of the study population. EUS‐HGS, endoscopic ultrasound‐guided hepaticogastrostomy; FCMS, fully covered metal stent; MBO, malignant biliary obstruction; PCMS, partially covered metal stent.

A total of 134 consecutive patients who underwent EUS‐HGS were identified in our database. After excluding 20 patients (technical failure, *n* = 11; benign stricture, *n* = 4; plastic stent, *n* = 5), 114 patients (FCMS, *n* = 50; PCMS, *n* = 64) were included for analysis. Matching both groups based on potential confounding factors using 1:1 propensity score matching, a total of 80 patients (40 FCMS, 40 PCMS) were selected (Fig. 3). The baseline characteristics of patients in the pre‐ and postmatched cohorts are presented in Tables 1 and 2. After matching, all the parameters were similar between both groups.

**Table 1:** Baseline characteristics of the patients

	Before propensity score matching				After propensity score matching			
	FCMS (*n* = 50)	PCMS (*n* = 64)	*P*‐value	SMD	FCMS (*n* = 40)	PCMS (*n* = 40)	*P*‐value	SMD
Mean age, years (SD)	63.1 (11.2)	68.6 (10.2)	0.008	0.505	65.9 (10.2)	66.9 (9.7)	0.653	0.101
Sex, M:F, *n* (%)	31 (62):19 (38)	35 (55):29 (45)	0.433	0.149	28 (70):12 (30)	26 (65):14 (35)	0.633	0.107
Mean age‐adjusted CCI (SD)	7.0 (2.2)	7.5 (2.1)	0.226	0.229	7.2 (2.3)	7.5 (2.1)	0.651	0.102
Indication for EUS‐HGS, *n* (%)			0.164	0.265			0.823	0.050
Primary EUS‐HGS	20 (40)	34 (53)			19 (48)	20 (50)		
Rescue EUS‐HGS	30 (60)	30 (47)			21 (53)	20 (50)		
Level of obstruction, *n* (%)			0.161	0.268			0.999	0.001
Proximal bile duct	36 (72)	38 (59)			28 (70)	28 (70)		
Distal bile duct	14 (28)	26 (41)			12 (30)	12 (30)		
Targeted LIHD, *n* (%)			0.243	0.222			0.999	0.001
B2	18 (36)	30 (47)			14 (35)	14 (35)		
B3	32 (64)	34 (53)			26 (65)	26 (65)		
Chemotherapy,^†^ *n* (%)			0.336	0.183			0.617	0.112
Yes	13 (26)	22 (34%)			12 (30)	10 (25)		
No	37 (74)	42 (66)			28 (70)	30 (75)		

^†^ A significant history of chemotherapy in the cohort was defined as having undergone at least two cycles of chemotherapy, regardless of the chemotherapy regimen, after endoscopic ultrasound‐guided hepaticogastrostomy (EUS‐HGS).

CCI, Charlson comorbidity index; F, female; FCMS, fully covered metal stent; LIHD, left intrahepatic duct; M, male; PCMS, partially covered metal stent; SD, standard deviation; SMD, standardized mean difference.

**Table 2:** Cause of malignant biliary obstruction

Cause	Unmatched cohort		Matched cohort	
	FCMS (*n* = 50)	PCMS (*n* = 64)	FCMS (*n* = 40)	PCMS (*n* = 40)
Cholangiocarcinoma	27 (54)	28 (44)	21 (53)	20 (50)
Pancreatic cancer	11 (22)	22 (34)	9 (23)	10 (25)
Hepatocellular carcinoma	3 (6)	0 (0)	3 (8)	0 (0)
Gallbladder cancer	2 (4%)	5 (8)	1 (3)	4 (10)
Other metastatic cancer	7 (14%)	9 (14)	6 (15%)	6 (15)

Values are *n* (%).

FCMS, fully covered metal stent; PCMS, partially covered metal stent.

### Technical/clinical outcomes and adverse events

The procedural outcomes of the two groups are summarized in Table 3. The technical success rate was 92% (123/134). After excluding patients with technical failure, the secondary outcomes were evaluated. In the matched cohort, the FCMS and PCMS groups showed a similar rate of clinical success (FCMS, 90% vs. PCMS, 88%; *P* = 0.999), early adverse event (15% vs. 20%; *P* = 0.556), late adverse event (18% vs. 33%; *P* = 0.121), and median overall survival (4.1 months [95% confidence interval (CI) 2.6–5.5] vs. 3.8 months [95% CI 1.9–5.7]; *P* = 0.609).

**Table 3:** Procedural outcomes of fully covered metal stents (FCMS) and partially covered metal stents (PCMS) for endoscopic ultrasound‐guided hepaticogastrostomy (HGS)

	Unmatched cohort			Matched cohort		
	FCMS (*n* = 50)	PCMS (*n* = 64)	*P*‐value	FCMS (*n* = 40)	PCMS (*n* = 40)	*P*‐value
Clinical success^†^	44/50 (88)	57/64 (89)	0.859	36/40 (90)	35/40 (88)	0.999
Early adverse event^†^	7/50 (14)	10/64 (16)	0.809	6/40 (15)	8/40 (20)	0.556
Late adverse event^†^	8/50 (16)	21/64 (33)	0.041	7/40 (18)	13/40 (33)	0.121
Stent occlusion^†^	7/50 (14)	20/64 (31)	0.032	6/40 (15)	12/40 (30)	0.108
Reintervention rate^†^	9/50 (18)	23/64 (36)	0.034	8/40 (20)	15/40 (38)	0.084
Percutaneous biliary drainage	1	3		1	2	
Stent‐in‐stent	2	14		2	7	
Stent change	6	0		5	0	
Additional HGS	0	4		0	4	
Embolization	0	1		0	1	
Balloon sweeping	0	1		0	1	
Patency rate, %			0.010^‡^			0.030^‡^
At 6 months	88.30%	59.70		85.20%	59.50	
At 12 months	75.20	41.80		75.80	43.40	
Follow‐up duration (days), mean (SD)	219.4 (213.7)	171.8 (139.4)	0.176	201.3 (195.6)	174.7 (150.2)	0.496

^†^Data are shown as n/N (%). ^‡^Log‐rank test.

SD, standard deviation.

In the FCMS group, early adverse events included transient fever (*n* = 4), hemobilia (*n* = 1), and sepsis (*n* = 1). One patient with sepsis was treated with additional biliary drainage using PTBD, and all the other patients with early adverse events showed improvement after conservative management. In the PCMS group, early adverse events included transient fever (*n* = 2), hemobilia (*n* = 1), sepsis (*n* = 3), and bile leakage (*n* = 2). In one patient with bile leakage, stent‐in‐stent placement of FCMS through the PCMS was performed to treat the adverse event. Except for this patient, all early adverse events in the PCMS group were treated with conservative management.

### Stent patency

**Figure 4:**
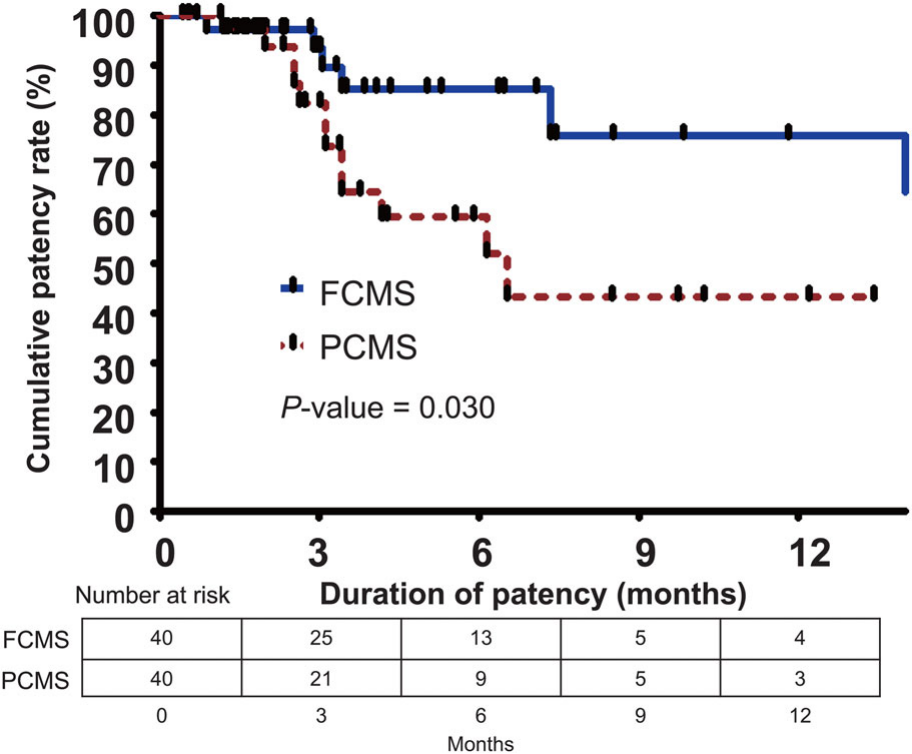
Kaplan–Meier curves of cumulative patency between fully covered metal stents (FCMS) and partially covered metal stents (PCMS) for endoscopic ultrasound‐guided hepaticogastrostomy.

In the matched cohort, both groups had a similar mean follow‐up duration (201.3 ± 195.6 days vs. 174.7 ± 150.2 days; *P* = 0.496). The stent occlusion rate (15% vs. 30%; *P* = 0.108) and reintervention rate (20% vs. 38%; *P* = 0.084) tended to be lower in FCMS when compared with PCMS, but there were no statistically significant differences. The cumulative stent patency is presented in Figure 4. The median cumulative patency in the FCMS group could not be calculated using the Kaplan–Meier method. The FCMS group showed a higher cumulative stent patency rate compared with the PCMS group (85.2% vs. 59.5% at 6 months; 75.8% vs. 43.4% at 12 months; *P* = 0.030, log‐rank test).

All instances of stent occlusion occurred as late adverse events. In the FCMS group, late adverse events included hemobilia (*n* = 1) and stent occlusion (*n* = 6). In the PCMS group, there was one case of pseudoaneurysm with bleeding, which was treated with embolization, and 12 cases of stent occlusion. In the FCMS group, all patients with stent occlusion underwent endoscopic reintervention; one patient underwent stent‐in‐stent placement of a plastic stent through the previous FCMS, and five patients underwent stent replacement through the previous hepaticogastric fistula. Meanwhile, patients with stent occlusion in the PCMS group underwent endoscopic management (stent‐in‐stent placement of plastic stent, *n* = 5; additional HGS through another route, *n* = 3; stent‐in‐stent placement of a metal stent, *n* = 1; balloon sweeping through the PCMS for stone removal, *n* = 1) and percutaneous biliary drainage (*n* = 2) to manage adverse events. The causes of stent occlusions were tissue overgrowth (*n* = 5 in FCMS), a combination of tissue ingrowth and/or overgrowth (*n* = 10 in PCMS), stone formation (*n* = 1 in FCMS; *n* = 1 in PCMS), and food reflux (*n* = 1 in PCMS).

## DISCUSSION

In this study, EUS‐HGS for unresectable MBO in the unmatched cohort showed high rates of technical (92%) and clinical success (89%), and an acceptable safety profile in terms of early adverse events (15%) observed with both FCMS and PCMS. These findings are consistent with the results of previous studies on EUS‐HGS, which reported technical success rates of 94–96%, clinical success rates of 85–92%, and early adverse event rates of 16–21%.^19^, ^20^, ^25^ The FCMS group had higher patency rates at 6 and 12 months compared to the PCMS group. In the long‐term analysis, the FCMS and PCMS groups showed comparable efficacy and safety. To the best of our knowledge, this study is the first to directly compare the outcomes between FCMS and PCMS for EUS‐HGS.

Despite the variations in terminology and definitions of adverse events, the stent occlusion rate (27/114, 24%) and reintervention rates (28%) in the unmatched cohort were comparable to those (stent occlusion rate, 13–37%; reintervention rate, 21–40%) reported in previous studies.^19^, ^20^, ^25^ In the present study, we found that the stent occlusion rate and reintervention rate were lower in the FCMS group than in the PCMS group in the unmatched cohort, while in the matched cohort, they tended to be lower in the FCMS group, without statistical significance. The lower stent occlusion and reintervention rates in the FCMS group may have contributed to the higher cumulative patency rate in the FCMS group. Despite the difference of stent patency between the two groups, there was no significant difference in terms of overall survival. This finding is aligned with previous studies showing that, while stent patency differ, overall survival remains comparable between FCMS and uncovered metal stent (UCMS) in the management of malignant biliary obstruction.^2^, ^26^

In a recent study comparing outcomes between FCMS and UCMS for ERCP in distal biliary obstruction, the stent patency is longer in FCMS than in UCMS.^26^ This study also reported a similar rate of stent overgrowth but higher stent ingrowth in UCMS compared with FCMS, suggesting that tumor ingrowth might induce higher stent occlusion and subsequently shorter stent patency in the UCMS group. Despite the different approach routes, the result regarding short stent patency in UCMS is similar to that of our study, considering that the uncovered portion of PCMS is placed in the intrahepatic duct. Although SEMS did not pass through the malignant stricture during EUS‐HGS stent ingrowth induced by hyperplasia of bile duct tissue can develop during indwelling PCMS, which was the most common reason for stent occlusion in both the current and previous studies.^9^ This suggests that the shorter stent patency in the PCMS group might be mainly due to stent ingrowth at the uncovered portion in the hepatic ends of PCMS. In theory, oversizing the stent diameter compared to intrahepatic duct (IHD) diameter may frequently induce tissue overgrowth as larger stents may induce more significant local inflammatory responses, potentially leading to hyperplasia and subsequent stent occlusion. However, in our clinical practice we did not specifically measure the IHD diameters to select the stent size, as the diameter of the IHD is not fixed and tends to decrease after decompression following stent insertion. Additionally, it is not practically feasible to precisely measure the maximal diameter of the IHD within the area covered by the uncovered portion of the metal stent using real‐time EUS. Nakai *et al*. also reported that the mismatch of diameter between PCMS and the bile duct did not affect cumulative time to recurrent biliary obstruction in EUS‐HGS.^9^ Further study investigating the optimal stent diameter in relation to tissue hyperplasia and its impact on long‐term patency is needed to provide guidelines for stent sizing to improve long‐term stent patency.

PCMS was expected to minimize adverse events associated with FCMS. However, none of the patients with FCMS in the unmatched cohort experienced stent migration, and there was no significant difference between FCMS and PCMS in terms of early adverse events. The stent design of FCMS, which includes antimigrating flares at both ends, might reduce the risk of migration. The choice of stent sizes in our study was based on clinical experience and institutional protocols. Our institution routinely uses an 8 mm diameter for PCMS and a relatively smaller diameter (6 mm) for FCMS when performing EUS‐HGS. In our experience, we found that the relatively small diameter (6 mm) of FCMS showed comparable efficacy when compared with larger diameters (8 mm), while minimizing the risk of side branch occlusion, although studies comparing the outcomes of each size of SEMS have not been conducted yet.^27^ In our study, the relatively smaller diameter (6 mm) of the FCMS may contribute to fewer adverse events, and there were no cases of blockage of segmental ductal branches. Although FCMS and PCMS are currently available in lengths ranging from 6 to 12 cm, we only used a 10 cm length of SEMS for EUS‐HGS, which is sufficient to prevent adverse events induced by stent migration toward the peritoneum. Previous studies have also recommended using SEMS with a length of more than 10 cm for EUS‐HGS to reduce the risk of stent migration into the peritoneal cavity.^9^, ^28^, ^29^ There may be concerns about the technical feasibility of endoscopic reintervention with longer stents; however, most patients, including those with PCMS, were able to undergo stent‐in‐stent placement.^9^

The methods of endoscopic reintervention varied, depending on the types of SEMS. When using the long stent for EUS‐HGS, it may be challenging to stabilize the scope position during stent‐in‐stent placement through the SEMS. This is the reason why we performed stent change through the HGS fistula instead of stent‐in‐stent placements in patients with FCMS occlusion. Meanwhile, various methods of endoscopic reintervention other than stent change, including stent‐in‐stent placement, balloon sweeping for stone removal, and additional HGS through another bile duct, were used in patients with PCMS occlusion because tissue ingrowth in the uncovered portion limited the removal of PCMS. Out of the 12 patients with stent occlusion in the PCMS group, two patients underwent percutaneous biliary drainage because the guidewire could not pass the obstructed site during endoscopic reintervention; in contrast, all patients with stent occlusion in the FCMS group underwent successful endoscopic reintervention. The different reintervention approaches in each type of stent can be one of the factors to consider when choosing the stent types for EUS‐HGS.

Our study has several limitations. First, the types of stents were chosen at the discretion of the endosonographer, which introduces selection bias. To enhance comparability and reduce selection bias, we conducted a propensity score matching analysis using confounding factors. Second, all procedures were performed by a highly experienced endosonographer in a high‐volume tertiary center. Therefore, the outcomes of EUS‐HGS may differ in other centers with varying procedural expertise. Third, the stent size was standardized within each group according to our institute's experience. Therefore, we were unable to assess the optimal size of SEMS for EUS‐HGS.

In conclusion, the use of FCMS and PCMS for EUS‐HGS in unresectable MBO showed similar rates of clinical success, early and late adverse events, and reintervention. However, FCMS showed a higher patency rate at 6 and 12 months compared to PCMS. Larger prospective studies are needed to confirm these results.

## CONFLICT OF INTEREST

Authors declare no conflict of interest for this article.

## FUNDING INFORMATION

None.

## ETHICS STATEMENT

Approval of the research protocol by an Institutional Reviewer Board: This study was approved by the Institutional Review Board of the Asan Medical Center (approval number: 2024–0290).

Informed Consent: N/A.

Registry and the Registration No. of the study/trial: N/A.

Animal Studies: N/A.

## Supporting information


**Data S1** Details of endoscopic ultrasound‐guided hepaticogastrostomy procedure.

**Video S1** Endoscopic ultrasound‐guided hepaticogastrostomy using partially covered metal stent.
