# Supplementary material for: Comparison of fully versus partially covered metal stents in endoscopic ultrasound‐guided hepaticogastrostomy for malignant biliary obstruction (with video)
Source: Dig Endosc. 2024 Nov 28;37(5):532–40. doi: 10.1111/den.14952 (PMC12062555; doi:10.1111/den.14952)
Supplement: Supplementary file 1 — Data S1 Details of endoscopic ultrasound‐guided hepaticogastrostomy procedure. [file DEN-37-532-s001.docx]

**Supplementary Methods**

**Procedures**

A single experienced endosonographer (T.J.S.) performed all procedures. Before the procedure, midazolam and meperidine were administered to provide conscious sedation for all patients. A conventional linear array echoendoscope (GF-UCT 260; Olympus Optical, Tokyo, Japan) was used for EUS-HGS. Before the needle puncture, a 0.025-inch guidewire (VisiGlide-2; Olympus, Tokyo, Japan) was preloaded into a 19-gauge needle with 3-way stopcock following the prefilling the 19-guage needle with normal saline. A 19-gauge needle (EUSN-19-T; Cook Endoscopy, Winston-Salem, NC, USA) punctured the dilated LIHD through the gastric wall. After guidewire was successfully advanced into the bile duct, contrast media was then injected into the dilated LIHD through the needle with 3-way stopcock to confirm biliary access under fluoroscopic guidance. When the cholangiogram was acquired, a guidewire was more advanced in the hilar direction. After removing the needle, a 4-mm biliary balloon dilator (REN, Kaneka Medical, Osaka, Japan) was inserted over the guidewire to dilate the fistula tract. Following tract dilatation, the FCMS or PCMS was advanced toward the LIHD over the guidewire. The intra-scope channel stent release technique was used to deploy the metal stent. If resistance was encountered during tract dilatation with REN balloon or the advancement of the metal stent, an additional tract dilatation was carefully performed using a 7 Fr Sohendra stent retriever (Cook Medical, Bloomington, IN, USA) or a 7 Fr cystotome (Taewoong Medical Co, Ltd, Goyang, South Korea) to facilitate the advancement of the stent.
